# Targeting Tyro3, Axl and MerTK (TAM receptors): implications for macrophages in the tumor microenvironment

**DOI:** 10.1186/s12943-019-1022-2

**Published:** 2019-05-14

**Authors:** Kayla V. Myers, Sarah R. Amend, Kenneth J. Pienta

**Affiliations:** 1 Department of Pharmacology and Molecular Sciences, The Johns Hopkins School of Medicine, Baltimore, MD USA; 2 The James Buchanan Brady Urological Institute, Department of Urology, The Johns Hopkins School of Medicine, Baltimore, MD USA; 3 Department of Oncology, The Johns Hopkins School of Medicine, Baltimore, MD USA; 4 Department of Chemical and Biomolecular Engineering, Johns Hopkins University, Baltimore, MD USA

**Keywords:** Macrophage, TAM receptors, Tyro3, Axl, MerTK, Efferocytosis, M2 macrophage polarization

## Abstract

Tumor-associated macrophages are an abundant cell type in the tumor microenvironment. These macrophages serve as a promising target for treatment of cancer due to their roles in promoting cancer progression and simultaneous immunosuppression. The TAM receptors (Tyro3, Axl and MerTK) are promising therapeutic targets on tumor-associated macrophages. The TAM receptors are a family of receptor tyrosine kinases with shared ligands Gas6 and Protein S that skew macrophage polarization towards a pro-tumor M2-like phenotype. In macrophages, the TAM receptors also promote apoptotic cell clearance, a tumor-promoting process called efferocytosis. The TAM receptors bind the “eat-me” signal phosphatidylserine on apoptotic cell membranes using Gas6 and Protein S as bridging ligands. Post-efferocytosis, macrophages are further polarized to a pro-tumor M2-like phenotype and secrete increased levels of immunosuppressive cytokines. Since M2 polarization and efferocytosis are tumor-promoting processes, the TAM receptors on macrophages serve as exciting targets for cancer therapy. Current TAM receptor-directed therapies in preclinical development and clinical trials may have anti-cancer effects though impacting macrophage phenotype and function in addition to the cancer cells.

## Background

Cancer is one of the leading causes of death worldwide. In the United States, 1,762,450 new cases and 606,880 cancer-related deaths are estimated to occur in 2019 [1]. Disease progression is strongly influenced by the tumor microenvironment (TME), comprised of all host cells and components beyond the cancer cells [2–5]. The TME is made of blood vessels, extracellular matrix (ECM) and many different host cell types including fibroblasts, B cells, and T cells. Tumor-associated macrophages are a critical component of the TME. In general, these macrophages have tumor-promoting functions such as tumor initiation, growth, angiogenesis, metastasis, immunosuppression and resistance to therapy [6]. Elucidating the role of macrophages in tumor biology is crucial as modulating their activity may open new opportunities for therapeutic interventions. Likewise, it is also important to understand how macrophages in the TME are impacted by current therapies aimed at targeting cancer cells.

Macrophages have diverse roles under normal physiologic conditions including phagocytosis, antigen-presentation and modulation of the immune response [7, 8]. There are different subtypes of macrophages that play unique and related roles in both normal and disease states. M2 macrophages support tissue homeostasis, wound-healing and resolution of inflammation. Tumor-associated macrophages are largely polarized to an alternatively-activated, M2-like state. In the TME, M2-like macrophages exert pro-tumor effects via their wound-healing functions. For example, M2 macrophages promote angiogenesis through secretion of pro-angiogenic factors [9, 10]. M2 macrophages suppress T cell infiltration and cytotoxic T cell function which impairs the anti-tumor immune response [11–13]. M2 tumor-associated macrophages also promote invasion and metastasis of cancer cells through ECM remodeling [14]. Another M2-associated function is phagocytosis of apoptotic cells, i.e., efferocytosis. Efferocytosis is enacted to resolve inflammation, repair tissue and suppress the immune system. In the TME, these effects promote tumor growth, metastasis and evasion of anti-tumor immunity [15, 16]. Thus, targeting tumor-associated macrophages and their function are potential therapeutic strategies due to their large influence in disease progression.

The TAM receptors (Tyro3, Axl and MerTK) are a well-studied family of receptors that, in addition to their function in many other cell types, including cancer cells, play roles in macrophage polarization and efferocytosis. The role of the TAM receptors in macrophages was first discovered upon the generation of TAM receptor single, double and triple knockout mice [17]. At approximately 4 weeks of age, the spleens and lymph nodes of the triple knockout mice were substantially larger compared to wild type (WT) mice due to hyperproliferation of constitutively active B and T lymphocytes. The triple knockout mice also developed autoimmune diseases such as rheumatoid arthritis and system lupus erythematosus [18]. These phenotypes were found to be due to altered macrophage and dendritic cell function. Following this work, the role of the TAM receptors on macrophages in different tissues and disease states has been studied. This review will summarize macrophage TAM receptor function in the context of the TME and discuss implications for interventions in cancer therapy.

## Main text

### The TAM receptor family and their ligands

**Fig. 1:**
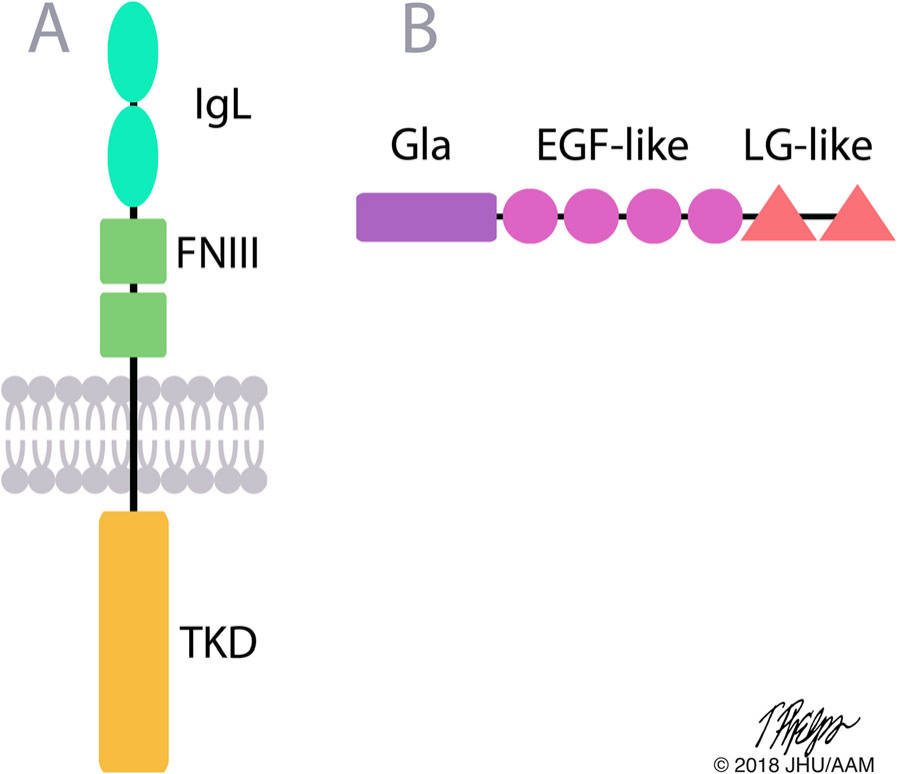
The structure of the TAM receptors and their shared ligands Gas6 and Protein S. **a** Tyro3, Axl and MerTK share a similar structure of two IgL domains, two FNIII domains and an intracellular TKD. **b** Gas6 and Protein S contain a Gla domain, four EGF-like domains and two LG-like domains. Abbreviations: IgL = immunoglobulin-like, FNIII = fibronectin type III, TKD = tyrosine kinase domain, Gla = γ-carboxyglutamic acid, EGF = epidermal growth factor, LG-like = laminin G

The TAM receptor family is comprised of three receptor tyrosine kinases that share similar structures distinct from other receptor tyrosine kinases (Fig. 1a). Each receptor’s extracellular domain contains two immunoglobulin-like (IgL) repeats and two fibronectin type III (FNIII) repeats. This is followed by a single helix transmembrane domain and the cytoplasmic tyrosine kinase domain (TKD) containing the KW(I/L)A(I/L)ES consensus sequence [19]. *Axl* was the first of this family discovered in studies identifying genes that transform NIH 3 T3 cells [20, 21]. MerTK was originally identified as the oncogene v-*ryk* from avian retroviruses and recognized as a member of the Axl family when the murine form was cloned [22, 23]. Tyro3 was the last of the three proteins to be added to the TAM receptor family based on its shared homology [24]. There are multiple alternative names for each in the published literature, and for clarity the names Tyro3, Axl and MerTK will be used throughout this review regardless of the naming convention used in the referenced literature. Axl is also known as UFO, Tyro7, JTK11 and ARK. MerTK is also referred to as MER, RP38, c-Eyk, c-mer, and Tyro12. Tyro3 can also be called RSE, BYK, Etk-2, Dtk, Rek, Sky and Tif.

The TAM receptors are activated upon binding of their extracellular ligands. Gas6 and Protein S were the first discovered and are the most studied ligands for the TAM receptors. *Gas6* was identified as one of the upregulated “growth arrest-specific” genes following serum starvation of NIH 3 T3 cells [25]. Gas6 was then confirmed in humans and recognized to have strong homology with Protein S [26, 27]. Protein S, also known as Pros1, is a Vitamin K dependent protein that has TAM receptor-independent roles in the blood coagulation cascade [28, 29]. As depicted in Fig. 1b, the amino terminus Gas6 and Protein S have a γ-carboxyglutamic acid (Gla) domain followed by four epidermal growth factor (EGF)-like repeats. Adjacent to the carboxy terminus are two laminin G (LG)-like domains that share sequence similarity to the sex hormone-binding protein (SHBP) [27]. Unique to Protein S is a thrombin sensitive cleavage site between the Gla and EGF-like domains [30]. While at first unclear, it is now understood that Gas6 binds all three receptors, whereas Protein S only activates Tyro3 and MerTK [27, 31–33]. There are three newly discovered ligands of the TAM receptors: Tubby and galectin-3, which bind MerTK, and Tubby-like protein 1 (Tulp-1) which binds all three receptors [34–36]. Due to the recentness of these TAM receptor ligand discoveries, not much as much information regarding their function is known compared to that of Gas6 and Protein S and this review will largely focus on the effects of ligands Gas6 and Protein S.

### TAM receptor-mediated signaling

Consistent with other receptor tyrosine kinases (RTKs), the TAM receptors become activated following ligand binding, receptor dimerization and subsequent trans-autophosphorylation of the kinase domains to activate intracellular signaling cascades and modulate gene transcription. More specifically, the TAM receptors are activated upon IgL domain binding to the LG-like domains of their ligand [37, 38]. Prior to activation, glutamic acid γ-carboxylation of the Gla domain the ligand Gas6 or Protein S is required [39].

The lipid membrane molecule phosphatidylserine (PtdSer) has been shown to strengthen ligand binding affinity and TAM receptor mediated signal transduction [40–43]. This interaction occurs when PtdSer binds to the Gla domain of Gas6 or Protein S in the presence of Ca^2+^ ions [40, 44]. In this context Gas6 and Protein S serve as bridging molecules for PtdSer and the TAM receptor. Adding PtdSer containing lipid membranes in the presence of TAM receptor ligand increases phosphorylation levels of TAM receptors compared to just adding ligand alone [41, 42]. This bridging interaction and signaling is important for phagocytosis of apoptotic cells exposing PtdSer.

There are a wide variety of complex downstream signaling pathways following TAM receptor phosphorylation. Detailed reviews summarizing known signaling pathways of individual TAM receptors have been published [19, 45, 46]. One of the most well characterized TAM receptor signaling pathways in macrophages is the phosphoinositide 3 kinase (PI3K)/Akt pathway. PI3K is made of a p85 regulatory subunit and a p110 catalytic subunit [47]. The intracellular kinase domain of phosphorylated Tyro3, Axl and MerTK can bind the p85 subunit of PI3K directly [48, 49]. Alternatively, the TAM receptors can also interact with p85 using growth factor receptor-bound protein 2 (Grb2) as a bridging protein [48, 50, 51]. Following activation of PI3K through either mechanism, PI3K phosphorylates Akt. In U937-derived macrophages, it has been shown that Gas6 mediated phosphorylation of Akt leads to glycogen synthase kinase 3β (GSK3β) phosphorylation and suppression of NF-kB nuclear translocation, thereby altering gene transcription [52]. The PI3K/Akt signaling axis has been shown to play roles in macrophage activation and polarization, suggesting a tole for the TAM receptors in regulating macrophage phenotype and function [53, 54].

### TAM receptor expression is influenced by macrophage polarization

Macrophages are influenced by cytokines and factors to adopt various phenotypes and functions. “Classically activated” M1 macrophages are involved in defense against bacteria and viruses and polarize in response to granulocyte-macrophage colony stimulating factor (GM-CSF), interferon-γ (IFN-γ) and microbial products such as lipopolysaccharide (LPS). These macrophages secrete pro-inflammatory cytokines and factors such as tumor necrosis factor α (TNF-α), interleukin-6 (IL-6), IL-1β, IL-12, IL-15, IL-18 and nitric oxide (NO). “Alternatively activated” M2 macrophages are stimulated by factors such as macrophage colony-stimulating factor (M-CSF), IL-4, IL-13, IL-10 and dexamethasone. Secreted products of M2 macrophages include transforming growth factor β (TGF-β), IL-10, IL-13 and IL-1α. Through their functions and cytokine production, M2 macrophages have roles in tissue homeostasis, wound healing and fighting parasitic infections. In the setting of the TME, M1 macrophages play anti-tumor roles that aid in tumor immunity and M2 macrophages have pro-tumor influences that support disease progression.

Monocytes are differentiated into macrophages which are then polarized to different subtypes. Upon monocyte to macrophage differentiation, MerTK expression is upregulated and Tyro3 levels remain unchanged [55–57]. There is conflicting evidence regarding Axl expression, with reports of both increased and decreased expression upon monocyte differentiation to macrophages [55, 56]. While Tyro3 mRNA and protein are expressed in monocytes and macrophages, there is a lack of literature characterizing Tyro3 expression levels on macrophages polarized to either an M1 or M2 subtype. However, Axl and MerTK expression on M1-like and M2-like macrophages has been described in the literature and will be summarized here.

Like the evidence for monocyte-to-macrophage differentiation, there is conflicting evidence in the literature regarding Axl expression on M1-like and M2-like macrophages. Several reports have demonstrated Axl expression to be higher on M2-like macrophages. Axl expression is increased in IL-4 and IL-13 polarized M2 macrophages compared to LPS and IFN-γ M1 polarized macrophages in murine bone marrow-derived macrophages (BMDMs) [58]. *AXL* transcripts levels are higher in M2-like human monocyte-derived macrophages polarized with M-CSF than M1-like polarized with GM-CSF [59]. In human monocyte-derived macrophages, *AXL* mRNA is induced following treatment with immunosuppressive M2-stimulant dexamethasone [56]. In contrast, however, there is also evidence that Axl expression is higher on M1-like macrophages. Following treatment with M1-stimulant LPS, BMDMs have increased Axl expression [41]. This study also observed Axl downregulation following dexamethasone treatment to induce M2-like macrophages. Pro-inflammatory viral triggers such as IFN-α and poly(I:C) also stimulate Axl expression on human monocyte-derived macrophages [41, 60]. Thus, it is possible that Axl expression on M1 versus M2 macrophages may differ model-to-model based on specific experimental conditions, such as source of monocytes and method of polarization.

MerTK expression has consistently been shown to be higher in immunosuppressive, M2-like macrophages than unstimulated and M1-like macrophages. It has been reported that *Mertk* mRNA transcript levels are higher in IL-4 and IL-13 M2 polarized BMDM’s than LPS and IFN-γ M1 polarized BMDM’s [58]. Monocyte-derived human macrophages treated with M2-stimulants M-CSF, IL-4, IL-10 or CD5L have higher MerTK protein levels than untreated macrophages or those stimulated with M1-stimulant GM-CSF, LPS, IFN-γ, IFN-α or poly (I:C) [60–63]. MerTK is also upregulated in human monocyte-derived macrophages following stimulation with dexamethasone and corticosteroids hydrocortisone and aldosterone, supporting MerTK expression in immunosuppressive, anti-inflammatory M2-like macrophages [41, 56, 60, 62, 64].

### TAM receptor signaling inhibits M1 polarization and induces M2 polarization

**Fig. 2:**
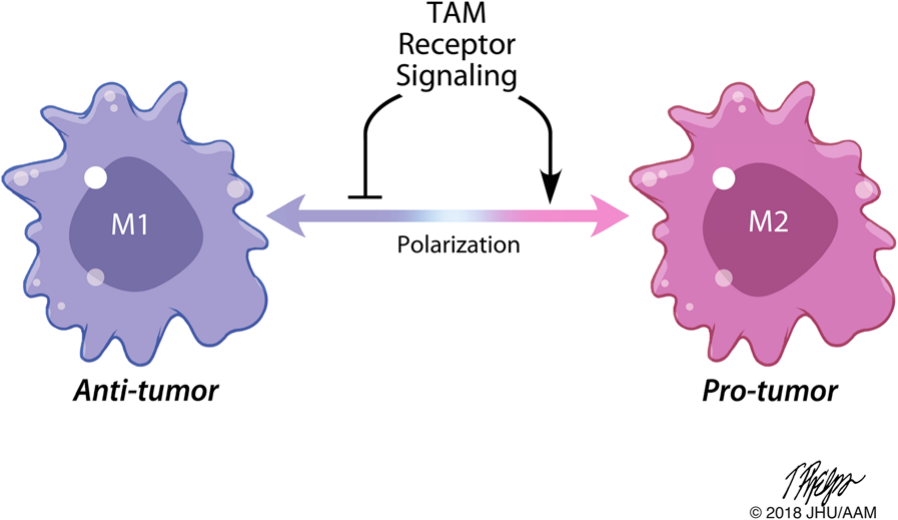
TAM receptor signaling skews macrophage polarization**.** TAM receptor binding and downstream signaling dampens M1 polarization and promotes M2 polarization. Overall, this decreases anti-tumor M1-like phenotypes and functions and increases pro-tumor M2-like phenotypes and functions

While LPS and IFN-γ are the standard M1 macrophage inducers and IL-4 and IL-13 are the standard M2 macrophage polarizing agents for in vitro polarization, there are many other proteins and stimulants that can skew macrophage polarization. TAM receptor signaling has been shown to play a role in shifting macrophage polarization by dampening M1 polarization and inducing M2 polarization (Fig. 2).

TAM receptor signaling has been shown to reduce M1 polarization, with most evidence showing a decrease in M1-associated cytokine secretion (e.g.; TNF-α, IL-6 and IL-1β) following TAM receptor activation by Gas6 or Protein S. In LPS-stimulated U937-derived macrophages, Gas6/MerTK signaling decreases secretion levels of TNF-α, IL-6 and IL-1β [52]. Similarly, Gas6/MerTK signaling inhibits TNF-α and IL-6 secretion in human monocytes/macrophages [52]. In an independent study, MerTK activation was shown to inhibit TNF-α production by LPS-stimulated mouse macrophages [65]. Additionally, exogenous Gas6 treatment on phorbol 12-myristate 13-acetate (PMA)-differentiated THP-1 macrophages decreases TNF-α, IL-1β and IL-6 mRNA and protein levels, providing further evidence that TAM receptor signaling blocks M1 polarization [66]. It has been shown that Protein S activation of either MerTK or Tyro3 inhibits LPS and IFN-γ induced M1 polarization of peritoneal and tumor-derived mouse macrophages, indicated by decreased expression of M1 marker genes *Il1*, *Il6*, *Cd86* and *Tnf* [67]. Stimulation of mouse peritoneal macrophages with Gas6 or Protein S decreases M1-associated inflammatory gene expression of IL-1β, IL-6 and TNF-α [68]. Additionally, inhibition of Gas6 or Protein S with neutralizing antibodies increases expression levels of these M1-associated pro-inflammatory genes [68]. In an invasive pulmonary aspergillosis (IPA) model, it has been shown that an anti-Axl antibody increases the number of M1 macrophages [69]. This was measured by an increase whole-lung *Inos* transcript levels, a marker of M1 activation and a decrease in M2 activation markers *Arg1* and *Fizz1*. Contradictory to these studies, inhibition of Axl with a monoclonal antibody has been shown to decrease M1-associated inflammatory factors IL-6, TNF-α and G-CSF production by tumor associated macrophages in MDA-MB-231 xenograft breast cancer models, suggesting Axl promotes M1 polarization some cases [70].

In addition to effects on M1 polarization, TAM receptor signaling has also been shown to promote pro-tumor M2 macrophage polarization. Gas6 mediated MerTK signaling in the RAW264.7 murine macrophage cell line increases mRNA and protein levels of M2-associated genes Arg2 and VEGF, as well as mRNA levels of *Arg1* [71]. Recently, it was discovered that mineral trioxide aggregate (MTA) polarizes THP-1 cells towards an M2 phenotype in an Axl signaling-dependent manner. The MTA-induced M2 polarization, indicated by increased M2 marker CD206 and M2-associated secreted cytokines IL-10, TGF-β and VEGF was blocked by the Axl small molecule inhibitor R428 [72]. M1 polarized THP-1 macrophages treated with Gas6 have increased protein levels of the M2 marker CD206 and mRNA levels of M2-associated genes *CD206* and *IL10* [73]. These macrophages also have decreased protein levels of M1 marker CD11b and increased STAT6 phosphorylation, an inducer of M2 polarization. Mice null for Galectin-3, a newly discovered MerTK ligand, have a reduced IL-4 and IL-13 induced M2 polarization, suggesting galectin-3 also skews macrophage polarization towards an M2-like phenotype [74]. Taken together, these data suggest that the TAM receptors play a role in shifting macrophage polarization away from an M1-like phenotype and towards an M2-like phenotype.

### Efferocytosis and M2-like polarization is tumor-promoting

A major role of macrophages is clearance of apoptotic cells, a process known as efferocytosis. Dendritic cells and specialized epithelial cells and fibroblasts also have the ability to efferocytose. This mechanism is enacted to maintain tissue homeostasis and prevent induction of inflammation by secondary necrosis. Under normal physiology, lack of efferocytosis is disadvantageous in many cases because secondary necrosis releases immunogenic components that can lead to chronic inflammation and autoimmune disorders [75]. However, in a cancer setting efferocytosis is pro-tumor as it induces a wound-healing immunosuppressive phenotype. Consistent with this phenotype, M2-like macrophages have a greater capacity for efferocytosis than M1-like macrophages [60, 76–79]. Due to its tumor-promoting role, the efferocytosis pathway is an exciting and new potential therapeutic target [16].

Efferocytosis is initiated by signals from the apoptotic cell that then promote cytoskeletal rearrangement and nuclear receptor signaling in the efferocytosing macrophage. “Find-me” signals promote attraction of monocytes and macrophages. Some well-established “find-me” signals are adenosine triphosphate (ATP), uridine triphosphate (UTP), lysophosphatidylcholine (LPC), fractalkine and spingosine 1-phosphate (S1P) which are released from the cell in a caspase-regulated manner during apoptosis [80–83]. Apoptotic cells also display “eat-me” signals on their surface for macrophages to tether to. The most characterized “eat-me” signal is the lipid moiety PtdSer. Healthy cells sequester PtdSer on the inner leaflet by action of flippase enzymes. During apoptosis, caspase activity inactivates the flippase adenosine triphosphatase type 11C (ATP11C) and activates the scramblase Xk-Related Protein 8 (Xkr8) allowing PtdSer exposure on the outer leaflet of the cell membrane [84, 85].

**Fig. 3:**
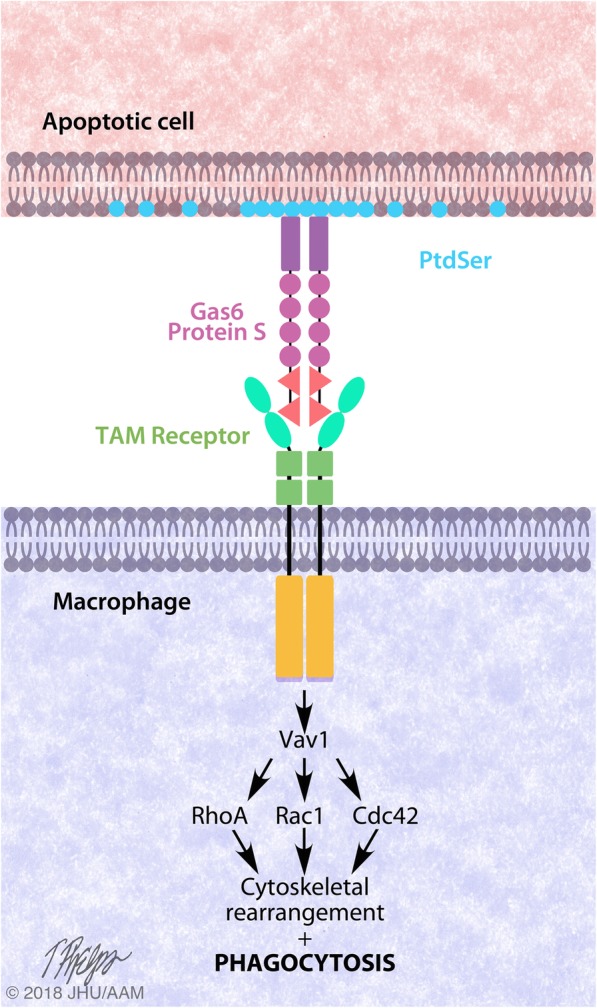
The TAM receptors mediate efferocytosis. The TAM receptors recognize phosphatidylserine (PtdSer) on the outer leaflet of apoptotic cell membranes using Gas6 and Protein S as bridging ligands. Ligand binding promotes TAM receptor dimerization and phosphorylation leading to Vav1-mediated activation of Rho GTPases RhoA, Rac1 and Cdc42. This signaling cascade induces a cytoskeletal rearrangement and phagocytosis of the apoptotic cell

The TAM receptors, among others such as phosphatidylserine receptor (PSR), CD36, Tim4, BAI1 and α_v_β_3_ integrin bind PtdSer to tether the macrophage to the apoptotic cell [86–91]. As discussed in a previous section, Gas6 and Protein S serve as bridging molecules between PtdSer and the TAM receptors (Fig. 3). PtdSer binds the Gla end of Gas6 and Protein S while the TAM receptors bind their LG domains. This allows macrophages to attach to the apoptotic cell prior to phagocytosis [92].

Following TAM receptor apoptotic cell tethering, cytoskeletal rearrangements induce phagocytosis. Evidence for the mechanism of cytoskeletal rearrangement by MerTK-mediated efferocytosis has been described. Following apoptotic cell binding, the phosphorylated tyrosine kinase domain of MerTK facilitates phosphorylation of Vav1 [93]. The binding of the cytoplasmic tail to Vav1 is phosphotyrosine-independent, although MerTK activation is required for Vav1 phosphorylation and release. Vav1 is a guanine nucleotide-exchange factor (GEF) that can stimulate guanosine diphosphate (GDP) to guanosine triphosphate (GTP) exchange. When phosphorylated, Vav1 activates Rho family members Rac1, Cdc42 and RhoA [93, 94]. These proteins temporally and spatially regulate cytoskeleton dynamics, which promote engulfment of the apoptotic cell [95–97].

**Fig. 4:**
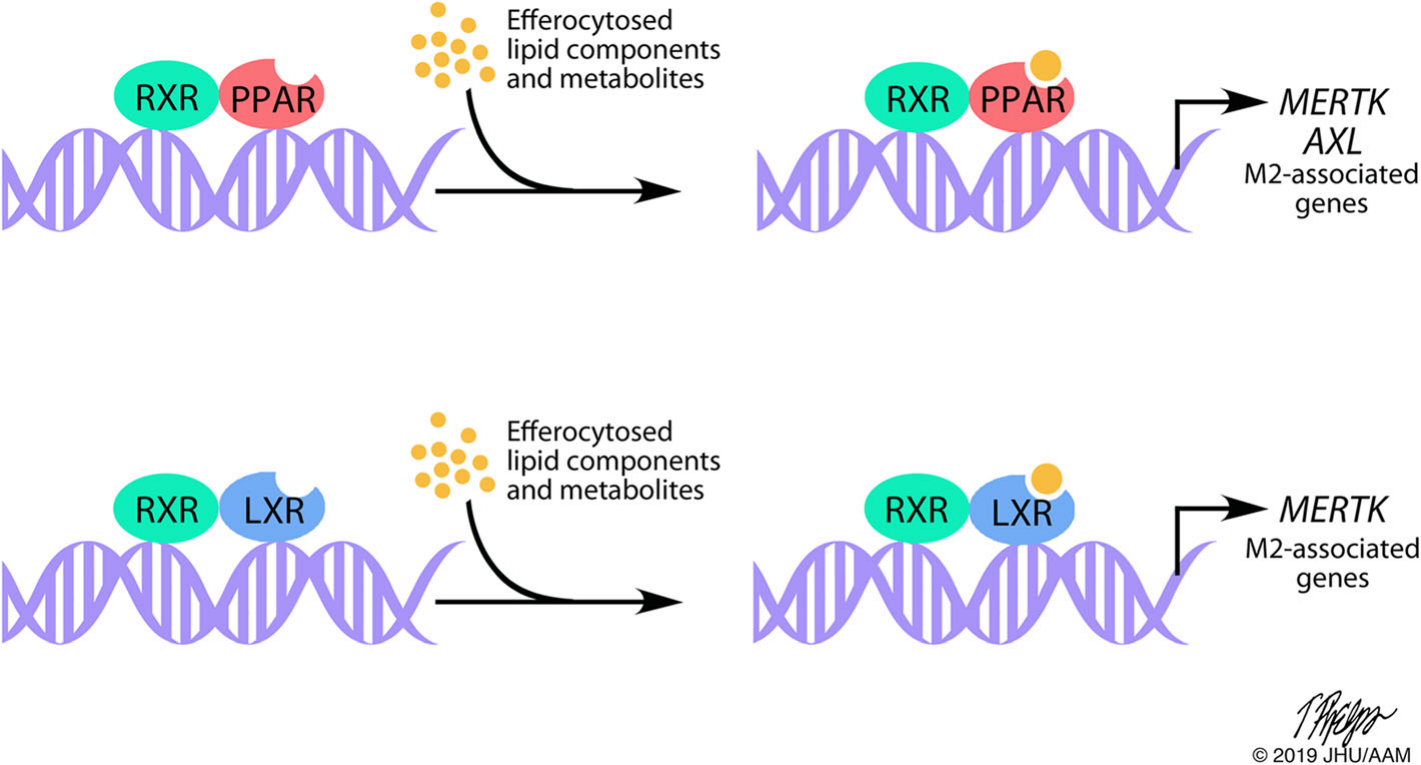
Nuclear receptors regulate gene transcription post-efferocytosis. PPARs and LXRs form heterodimers with RXRs to regulate gene transcription. Following efferocytosis, lipid components and metabolites from the apoptotic cell bind PPARs and LXRs. These activated nuclear receptors act as transcription factors to upregulate *MERTK*, *AXL* and M2-associated gene expression

Following engulfment of apoptotic cells, nuclear receptors are engaged in response to an increase in metabolic demand from the ingested cellular components (Fig. 4). Specifically, retinoid X receptors (RXRs) form heterodimers with peroxisome proliferator activated receptors (PPARs), and liver X receptors (LXRs) that have important roles in efferocytosis and other macrophage functions [98, 99]. These nuclear receptors have been shown to be associated with transcription of genes controlling and associated with M2 polarization [100–104]. This evidence suggests that nuclear receptor signaling following efferocytosis further polarizes macrophages to an M2-like phenotype. Notably, the process of efferocytosis itself promotes increased secretion of immunosuppressive cytokines associated with M2-like macrophages such as IL-10, TGF-β, IL-4 [105–107]. Apoptotic cell clearance by macrophages also decreases secretion of immune-stimulating cytokines associated with M1-like macrophages such as TNF-α, IL-1β and IL-12 [105, 107]. This evidence indicates that efferocytosis further promotes M2 polarization and function that can drive disease progression.

Nuclear receptors also regulate gene expression of machinery necessary for efferocytosis such as *MERTK* and *AXL*. PPAR-δ, PPAR-γ and RXRα have been shown to increase *MERTK* and *AXL* transcription in macrophages [108–111]. Pharmacologic inhibition or knockout of these nuclear receptors impairs apoptotic cell phagocytosis. However, in a separate study, MerTK and Gas6 expression was shown to increase with a PPAR-γ antagonist [112]. This conflicting evidence may reveal a further layer of MerTK regulation by nuclear receptors depending on the macrophage phenotype. Following apoptotic cell phagocytosis, LXR signaling is also induced [113]. *MERTK*, but not *TYRO3* or *AXL*, is a direct target gene of LXR nuclear receptors. As a result, *MERTK* transcription is increased following efferocytosis, creating a positive feedback loop. It has also been shown that MerTK signaling increases LXR abundance, which could potentiate this loop further [114].

One of the non-cytokine secreted products following apoptotic cell clearance by macrophages is hepatocyte growth factor (HGF) [115]. Shown in RAW 264.7 cells, this increase in HGF production was found to be mediated by MerTK (but not Axl or Tyro3) activation via RhoA-mediated signaling [116, 117]. Gas6/MerTK stimulated HGF production was found involve the same signaling pathway and was able to stimulate wound repair and cell growth of LA-4 epithelial cells [118]. While these studies speculated implications with alveolar macrophages, it remains of interest if these findings can translate to tumor associated macrophages since HGF can promote disease through HGF/c-Met signaling in a number of cancers [119].

While all three TAM receptors mediate efferocytosis in macrophages MerTK has been found to be essential to the process. MerTK was first identified as a phagocytotic receptor when it was observed that *Mertk* deficient mice had dramatically reduced clearance of apoptotic thymocytes [120]. While apoptotic cell clearance by macrophages is nearly abolished in *Mertk* deficient mice, it is also substantially decreased in *Axl* and *Tyro3* single knockout mice [57]. Additionally, RAW264.7 macrophages treated with an anti-MerTK antibody have impaired phagocytosis of apoptotic thymocytes [113]. Although Axl and Tyro3 mediate efferocytosis in macrophages, they may play more dominant roles in efferocytosis by dendritic cells [57, 121]. It has also been proposed that macrophage efferocytosis is dominated by MerTK in an immunosuppressive setting, whereas Axl is important in an inflammatory setting, dominating efferocytosis [41].

### TAM receptor inhibition as a therapy for cancer

Given what is known about the role of the TAM receptors in macrophages, key members of the TME, TAM receptor inhibition is a strong candidate for an anti-cancer therapy. TAM receptor inhibition may impair pro-tumor effects of M2-like macrophages by blocking efferocytosis. Since TAM receptor activation also skews macrophages polarization from an M1 to M2 phenotype, TAM receptor inhibition would decrease M2 characteristics of tumor-associated macrophages. As M2 macrophages have tumor-promoting roles involving cancer progression and immune suppression, impairing M2 polarization will slow disease progression. In addition to dampening M2 polarization, TAM receptor inhibition will increase polarization of M1-like macrophages in the TME. Since M1 macrophages have anti-tumor functions, shifting the balance of macrophages towards an M1 phenotype is beneficial in cancer. For example, in ovarian cancer the ratio of M1 to M2 tumor-associated macrophages decreases with increasing cancer stage, and a high ratio correlates with increased survival [122]. Similarly, pediatric classical Hodgkin Lymphoma patients with a predominant M1 polarization have a better overall survival [123].

There have been a few studies showing that MerTK inhibition, specifically on macrophages, is an effective anti-cancer therapy. In a number of different cancer types and tumor models, there is increased tumor macrophage infiltration following radiation therapy and these macrophages have been suggested to confer radioresistance to cancer cells [124–126]. MerTK on tumor associated macrophages has been shown to play a role in radiation therapy resistance. Following radiation therapy in a colorectal cancer mouse model, MerTK, Protein S and Gas6 are upregulated in tumor associated macrophages [127]. Neither Axl nor Tyro3 expression are changed. In this study *Mertk* knockout mice had a stronger overall survival following radiation therapy than wild type mice. This suggests that targeting MerTK on tumor associated macrophages may impair resistance following radiation therapy.

Inhibition of MerTK on leukocytes decreases tumor growth and metastasis in in vivo models of breast cancer, melanoma and colon cancer [128]. *Mertk* knockout mouse had decreased levels of the wound-healing cytokine IL-10 and increased levels of inflammatory cytokines IL-12 and IL-6 levels. Specific to the CD11b + macrophage population, IL-6 expression was increased compared to the WT mice. These changes in cytokine expression indicates a more M1-like macrophage phenotype. These *Mertk* knockout mice also had higher levels of CD8+ T cells following tumor inoculation, suggesting the change in cytokine expression may help promote anti-tumor immunity. This suggests that targeting MerTK on leukocytes, including macrophages, has multiple arms of anti-cancer activity.

In addition to impacting macrophage polarization and function, the TAM receptors have been shown to drive disease progression through their direct roles in growth, migration and therapy resistance of cancer cells. In certain cell line and mouse models for glioma, Kaposi sarcoma, mesothelioma, and non-small cell lung cancer, Axl inhibition decreases tumor growth [129–132]. Tyro3 is a proposed target in ovarian cancer due to its role in taxol resistance [133]. Additionally, siRNA silencing of Tyro3 has been shown to reduce proliferation in breast cancer cell lines [134, 135]. In non-small cell lung cancer, MerTK expression correlates with chemotherapy resistance, proliferation and migration [132, 136]. Thus, for some cancers the TAM receptors may serve as a strong dual target to impair both the tumor cells as well as tumor-associated macrophages.

Due to similarities in structure in the three TAM receptors, it is difficult to develop inhibitors specific for a single TAM receptor. Some TAM receptor inhibitors have specificity for other RTKs. However, targeting multiple TAM receptors may be advantageous as MerTK upregulation as a result of silencing or inhibiting Axl has been observed in several cancer models, suggesting the MerTK mediates resistance to treatments targeting Axl [137]. Additionally, as all three TAM receptors mediate M2 polarization and efferocytosis by macrophages, targeting multiple TAM receptors may have a stronger anti-cancer effect.

R428 is a small molecule inhibitor of Axl widely used in preclinical studies and in a number of clinical trials (see Table 2). While this inhibitor most potently inhibits Axl, there is off-target inhibition of other kinases including MerTK and Tyro3 [138]. R428 has demonstrated anti-cancer activity in a number of preclinical studies. For example, R428 decreases invasion and growth of the esophageal adenocarcinoma cell line OE33, as well as increases sensitivity to Lapatinib [139]. This inhibitor also induces apoptosis in freshly isolated B-cell chronic lymphocytic leukemia and at high doses overcomes stroma-mediated protection [140]. R428 blocks growth and migration of erlotinib resistant tongue squamous cell carcinoma HN5 cells [141]. Axl is overexpressed in metformin resistance prostate cancer LNCaP cells and Axl inhibition with R428 re-sensitized these resistant cells to metformin [142]. In vivo, R428 slows tumor growth of mice subcutaneously injected with chronic myeloid leukemia (CML) cell lines [143]. It has also been shown that R428 in combination with paclitaxel reduces proliferation of uterine serous cancer xenograft models [144]. While these studies focused on the effect of R428 on cancer cells and disease progression, it remains unknown if R428 inhibition of Axl on macrophages contributed to the positive results in these studies. Other Axl inhibitors are currently being used in preclinical studies and demonstrate therapeutic effects utilizing in vitro and in vivo cancer models (see Table 1).

**Table 1:** Summary of TAM receptor inhibitors in preclinical studies

TAM Targeting Drug (Drug Type)	Target(s)	Outcomes in cancer models
2,4-diaminopyrimidine-5-carboxamide analogs (Small molecule)	**Tyro3**	Not reported.
“Compound 47” (Small molecule)	**Tyro3**, IGF-1R, EphA2	Anti-cancer properties in HCC cell lines and xenograft models [158].
DP-3975 (Small molecule)	**Axl**	Anti-cancer properties in mesothelioma cell lines [131].
GL21.T (RNA aptamer)	**Axl**	Anti-cancer properties in a glioblastoma and a lung cancer cell line and mouse models [164].
Mer590 (Monoclonal antibody)	**MerTK**	Anti-cancer properties in lung cancer cell lines [157].
NPS-1034 (Small molecule)	**Axl**, Met	Anti-cancer properties in EGFR inhibitor resistant lung cancer cell lines and xenograft models [165].
Spiroindoline-based analogs (Small molecule)	**Tyro3**	None reported.
UNC569 (Small molecule)	**MerTK, Axl, Tyro3**	Anti-cancer properties in ALL and AML cell lines [146, 147].
UNC1062 (Small molecule)	**MerTK**	Anti-cancer properties AML cell lines [147].
UNC1666 (Small molecule)	**MerTK**, Flt3	Reduces colony formation in AML cell lines [148].
UNC2025 (Small molecule)	**MerTK**, Flt3	Anti-cancer properties in non-small cell lung cancer, leukemia and glioblastoma mouse models [152–155].
UNC 2250 (Small molecule)	**MerTK**	Anti-cancer properties in mantle cell lymphoma cell lines and mouse models [166].
UNC2541 (Small molecule)	**MerTK**	None reported.
YW327.6S2 (Monoclonal antibody)	**Axl**	Anti-cancer properties in lung and breast cancer mouse models [70].

TAM receptor-specific targets are bolded

Several MerTK inhibitors are also in development (see Table 1). UNC569, UNC1062, UNC1666, UNC2025 and UNC2250 are MerTK small molecule inhibitors that have demonstrated anti-cancer activity in preclinical models [145–150]. UNC569 induces apoptosis, improves sensitivity to cytotoxic chemotherapy and reduces colony formation in ALL cell lines [146]. UNC569 has also been utilized in vivo to study phagocytosis by retinal pigment epithelium cells [151]. UNC1062, as well as UNC569, induces apoptosis and decreases cell growth in AML cell lines [147]. UNC1666 is a dual MerTK and FLT3 inhibitor that reduces colony formation in MerTK- or FLT3-ITD- expressing AML cell lines [148]. UNC1062 and UNC1666 have poor oral bioavailability, so their clinical utility may be limited [148, 149]. UNC2025 is an orally bioavailable dual MerTK and FLT3 inhibitor [149]. In preclinical studies it has been demonstrated to have therapeutic effects in non-small cell lung cancer, leukemia and glioblastoma models [152–155]. Interestingly, one study focused on glioma-associated macrophages and microglia and found that UNC2025 alone and in combination with radiotherapy decreased the number of CD206+ M2 macrophages [155]. This supports the hypothesis that blocking M2 polarization via targeting TAM receptor signaling may impair disease progression. More recently, a macrocyclic pyrimidine, UNC2541, was synthesized and found be selective for MerTK over the other TAM receptors and FLT3 [156]. Mer590, a monoclonal antibody against MerTK, decreased MerTK expression in several lung cancer cell lines as well as increased apoptosis, sensitivity to carboplatin and decreased colony formation [157].

Although less studied, synthesis of Tyro3 inhibitors has also been explored (see Table 1). E/Z 6-Chloro-3-(3-trifluoromethyl-benzyliden)-1,3-dihydroindol-2-one (“Compound 47”) has been shown to block Tyro3 phosphorylation HuH7 cells [158]. Additionally, spiroindoline-based and 2,4-diaminopyrimidine-5-carbox-amide inhibitors of Tyro3 have been made [159–161].

Small molecule inhibitors, antibody-drug conjugates, Axl-Fc fusion proteins and CAR-T therapies for the TAM receptors are in clinical trials. Summarized in Table 2, most clinical trials have primarily aimed to target Axl because of Axl’s known role in driving disease progression through expression on cancer cells, although some inhibitors also inhibit MerTK and other RTKs with lower affinity. Active trials are in Phase I or II with some trials studying combinational effects with other cancer treatments. A Phase I clinical trial of ASLAN-002 with published results has been completed, and it was determined that 300 mg twice daily is a well-tolerated dose and is recommended for Phase II [162]. The most commonly reported adverse events were nausea, fatigue and constipation, and atrial fibrillation was reported as a dose limiting toxicity. Cabozantinib is a multi-kinase small molecule inhibitor that targets Axl, c-Met, VEGFR2, RET, KIT, and FLT3. This therapy is FDA-approved for advanced renal cell carcinoma, hepatocellular carcinoma and medullary thyroid cancer. The most commonly reported adverse events for this inhibitor are diarrhea, palmar-plantar erythrodysesthesia, fatigue, nausea, decreased appetite, hypertension, vomiting, weight loss, and constipation [163].

**Table 2:** Summary of clinical trials targeting TAM receptor activity

TAM Targeting Drug (Drug Type)	Target(s)	Condition(s)	ClincialTrials.gov Identifier(s)
AVB-S6–500 (Axl-Fc fusion protein)	**Gas6**	Ovarian Cancer Epithelial Ovarian Cancer Primary Peritoneal Carcinoma Fallopian Tube Cancer	NCT03401528 NCT03607955 NCT03639246
ASLAN-002/BMS-777607 (Small molecule)	Met, RON, FLT3, **Axl**	Advanced or Metastatic Solid Tumors	NCT00605618 NCT01721148 [162]
BA3011/CAB-AXL-ADC (Antibody-drug conjugate)	**Axl**	Solid Tumor Non-Small Cell Lung Cancer Castration-Resistant Prostate Cancer Pancreatic Cancer	NCT03425279
Bemcentinib/BGB324/R428 (Small molecule)	**Axl**	Advanced or Metastatic Solid Tumors	NCT02424617 NCT02488408 NCT02872259 NCT02922777 NCT03184558 NCT03184571 NCT03649321 NCT03654833
BPI-9016 M (Small molecule)	**Axl**, Met	Solid Tumors Non-Small Cell Lung Cancer	NCT02478866 NCT02929290
CCT301 (CAR-T)	**Axl**	Renal Cell Carcinoma	NCT03393936
INCB081776 (Small molecule)	**Axl**, **MerTK**	Advanced Solid Tumors	NCT03522142
MRX-2843 (Small molecule)	**MerTK**, FLT3	Advanced or Metastatic Solid Tumors	NCT03510104
ONO-7475 (Small molecule)	**Axl**, **MerTK**	Advanced or Metastatic Solid Tumors Acute Leukemia	NCT03176277 NCT03510104
TP-0903 (Small molecule)	**Axl**	Advanced Solid Tumors Chronic Lymphocytic Leukemia Small Lymphocytic Lymphoma EGFR Positive Non-small Cell Lung Cancer Colorectal Carcinoma Recurrent Ovarian Carcinoma BRAF-Mutated Melanoma	NCT02729298 NCT03572634

TAM receptor-specific targets are bolded. Where available, published results are cited

## Conclusions

When a drug is administered, all of the cells in the microenvironment are impacted by the drug, not just the cancer cells. While current clinical trials are aimed at targeting the tumor cells, it is of interest if targeting the macrophages will contribute to the results of these trials. Given the strong evidence that the TAM receptors on macrophages have tumor-promoting roles of promoting M2 polarization and efferocytosis, it is possible that targeting the TAM receptors on macrophages will be an effective therapy for treating different types of cancers. More preclinical evidence and clinical studies with a focus on macrophages could help determine the therapeutic relevance of targeting the TAM receptors as a cancer treatment.

## Abbreviations

ATP: Adenosine triphosphate
ATP11C: Adenosine triphosphatase 11C
BMDM: Bone marrow-derived macrophages
CML: Chronic myeloid leukemia
ECM: Extracellular matrix
EGF: Epidermal growth factor
FNIII: Fibronectin type III
GDP: Guanosine diphosphate
GEF: Guanine nucleotide-exchange factor
Gla: γ-carboxyglutamic acid
GM-CSF: Granulocyte-macrophage colony-stimulating factor
Grb2: Growth factor receptor-bound protein 2
GSK3β: Glycogen synthase kinase 3β
GTP: Guanosine triphosphate
HGF: Hepatocyte growth factor
IFN: Interferon
IgL: Immunoglobulin-like
IL: Interleukin
IPA: Invasive pulmonary aspergillosis
LG: Laminin G
LPC: Lysophosphatidylcholine
LPS: Lipopolysaccharide
LXR: Liver X receptor
M-CSF: Macrophage colony-stimulating factor
MTA: Mineral trioxide aggregate
NO: Nitric oxide
PI3K: Phosphoinositide 3 kinase
PMA: Phorbol 12-myristate 13-acetate
PPAR: Peroxisome proliferator activated receptor
PSR: Phosphatidylserine receptor
PtdSer: Phosphatidylserine
RTK: Receptor tyrosine kinase
RXR: Retinoid X receptor
S1P: Spingosine 1-phosphate
SHBP: Sex hormone binding protein
TAM Receptors: Tyro3, Axl and MerTK
TGF-β: Transforming growth factor β
TKD: Tyrosine kinase domain
TME: Tumor microenvironment
TNF: Tumor necrosis factor
Tulp-1: Tubby-like protein 1
UTP: Uridine triphosphate
WT: Wild type
Xkr8: Xk-Related Protein 8
